# Sea Cucumber Collagen Peptides Exert an Anti-Skin Aging Effect by Inhibiting Endoplasmic Reticulum Stress in Fibroblasts

**DOI:** 10.3390/foods15071147

**Published:** 2026-03-27

**Authors:** Rui Mi, Biyi Chen, Juncai Leng, Wei Zhao, Shan Gao, Jingwei Jiang, Jing Lan, Zunchun Zhou

**Affiliations:** 1 Key Laboratory of Germplasm Improvement and Fine Seed Breeding for Marine Aquatic Animals, Liaoning Ocean and Fisheries Science Research Institute, Dalian 116023, China; lianyi7432@sina.com (R.M.); cbysdsu2020@163.com (B.C.); gs_7920@163.com (S.G.); weijingjiang@live.cn (J.J.); 2 School of Food Science and Technology, Jiangnan University, Wuxi 214122, China; lengjuncai@163.com; 3 Dalian Fengyuan Biotechnology Company Limited, Dalian 116033, China; zhenjiumarketing@163.com

**Keywords:** sea cucumber collagen polypeptides, skin aging, ER stress, fibroblasts

## Abstract

Skin aging is a complex biological process triggered by intrinsic and extrinsic factors, causing structural and functional deterioration, and its mitigation is a priority in cosmetology and functional food science. Skin fibroblasts, which mediate skin repair, wound healing and inflammation, are closely associated with aging. Sea cucumber collagen peptides exhibit prominent anti-aging, immunomodulatory and antioxidant properties, yet their mechanisms in ameliorating skin aging remain elusive, necessitating further exploration. This study verified the anti-skin aging efficacy of sea cucumber collagen peptides in D-galactose-induced aging mice, and explored whether the mechanism involves regulating endoplasmic reticulum (ER) stress in skin fibroblasts. Aging mice were gavaged with sea cucumber collagen peptides; skin moisture, barrier function and hydroxyproline content were measured, and skin morphology was observed. Immunofluorescence and Western Blot were used to detect ER stress-related proteins. Results showed that sea cucumber collagen peptides significantly improved aging mouse skin barrier function, elevated water and collagen fiber contents, and ameliorated the status of fibroblasts and prickle cells. The underlying mechanism may involve inhibiting ER stress in skin fibroblasts and enhancing prickle cell function. These findings confirm the peptides’ high bioavailability and potential as anti-aging functional food ingredients, providing insights for skin aging prevention.

## 1. Introduction

The skin ranks as the largest organ in the human body, forming a protective barrier on the surface of the body and playing an important protective role. It exhibits multiple physiological functions, including protection, immunity, sensation, and neuroendocrine regulation [1]. Skin and mucosal tissues are the main gateways for the invasion of pathogenic microorganisms and foreign particles. Prickle cells, as an important component of the skin immune system, play an important role in improving skin barrier function and maintaining human health. The desmosomal junctions between stratum spinosum cells can prevent water loss, maintaining a certain level of skin moisture. They exert an effect in preserving the structural integrity of the skin, providing mechanical support, protecting the skin from external damage, and participating in cell proliferation and differentiation [2]. Aging is a complex process that involves the progressive decline of multiple tissue functions, leading to an increased probability of death [3]. Skin aging is a complex biological process involving multiple internal and external factors [4], ultimately leading to the degradation of skin structure and function [5]. The mechanism of anti-skin aging treatment mainly relies on enhancing the structure and function of the skin and addressing various biological changes that occur during the aging process [6]. Skin fibroblasts located in the dermal layer are the main cells of the skin matrix and perform important functions [7]. They not only participate in the structural construction of the skin but also play a crucial role in processes including wound healing, inflammatory response, aging, and so on [8,9]. Skin fibroblasts are responsible for synthesizing and secreting the major components of the skin matrix, including collagen, elastin, and glycosaminoglycans (GAGs) such as hyaluronic acid, chondroitin sulfate, and heparan sulfate [10]. GAGs and proteoglycans (PGs) together form the extracellular matrix (ECM) of the skin, where they contribute to structural integrity, regulate cellular signaling, and provide lubrication and buffering capacity to maintain skin hydration and resistance to compression [11]. Matrix metalloproteinases (MMPs) are capable of degrading nearly all components of the extracellular matrix [12]. Among them, MMP-1 and MMP-3 are specifically involved in the degradation of matrix components. At moderate levels, these enzymes help maintain skin structure and support tissue renewal, and they are also synthesized and secreted by fibroblasts [13]. Fibroblasts continuously produce new matrix components, thereby preserving the mechanical strength and integrity of the skin. However, during skin aging, fibroblast function declines, resulting in reduced skin elasticity and the formation of wrinkles [14]. Fibroblasts regulate skin metabolism to maintain ECM homeostasis and modulate fibrosis [15]. In addition, fibroblasts secrete cytokines and chemokines such as IL-6, TNF-α to participate in the skin immune response, and regulate skin immune function [16]. As organisms age, so does the skin, and collagen synthesis decreases, leading to loss of skin elasticity and the appearance of wrinkles; the activity of matrix metalloproteinases increases, leading to accelerated degradation of collagen and elastin in the skin, further promoting skin aging. The proliferative capacity of aging fibroblasts weakens, causing a decrease in skin repair ability [17].

Cellular senescence of fibroblasts is also closely associated with endoplasmic reticulum (ER) stress, which refers to the buildup of unfolded or misfolded proteins within the ER [18]. This accumulation provokes a sequence of stress-related reactions collectively called the unfolded protein response (UPR) [19]. The UPR is composed of three major signaling cascades, each set in motion by transmembrane protein sensors anchored in the ER membrane: IRE1α, PERK, and ATF6α [20]. Glucose regulated protein 78 (GRP78) is a companion heat shock protein that helps unfolded proteins fold or degrade correctly [21]. The UPR and ER stress initially serve as a mechanism to protect cells by restoring normal function [22]. However, prolonged and severe ER stress, whether continuous or intensified, results in cell dysfunction or even death [23]. The decreased activity of age-related key molecular chaperones and folding enzymes can impair the correct folding of proteins and trigger ER stress [24]. Alleviating ER stress may be an important strategy for slowing down skin aging [25]. Therefore, studying and improving the function of fibroblasts may provide an effective intervention strategy for skin aging.

Sea cucumber, as a marine organism, has long held an important position in human diet and traditional medicine [26]. With the advancement of scientific research technology, the diverse efficacies and potential health benefits of sea cucumber have been gradually explored in depth, particularly exhibiting significant potential in anti-aging [27], antioxidation [28], immune regulation [29], anti-tumor activity [30], cell proliferation and tissue repair [31], and insulin resistance amelioration [32]. Research indicates that sea cucumber polypeptides can extend the lifespan of *Caenorhabditis elegans* via DAF-16/DAF-2/SOD-3/OLD-1/PEPT-1 [33]. Sea cucumber polypeptides also have the effect of prolonging the lifespan of fruit flies and mice [27]. Furthermore, sea cucumber polypeptides can promote the proliferation of fibroblasts, upregulate the expression of type I collagen and tissue inhibitor of metalloproteinase-1 (TIMP-1), inhibit the expression of MMP-1, increase collagen secretion, and delay skin aging. Sea cucumbers have broad application prospects in the fields of medicine and nutrition. Although existing experimental data provide a solid theoretical foundation for the health benefits of sea cucumbers, current research is still in the exploratory stage.

This study established a D-galactose-induced aging mouse model, prepared collagen peptides from sea cucumbers, and administered them to aging mice via gavage for 4 weeks. Skin moisture content and skin barrier function were evaluated. Immunofluorescence, H&E staining, and electron microscopy were used to analyze related gene expression, protein levels, and tissue structural changes. The purpose of this study was to investigate the ameliorative effects and underlying mechanisms of sea cucumber collagen peptides against skin aging, thereby providing a theoretical foundation for the development of medicinal and edible anti-aging products.

## 2. Materials and Methods

### 2.1. Experimental Materials

The experimental sea cucumbers were purchased from Dalian Haixingdao Food Co., Ltd., Dalian, China, which were wild-caught individuals from the Dalian region. The molecular weights were detected by high performance liquid chromatography (HPLC) (Agilent Technologies, Santa Clara, CA, USA). The morphology and microstructure of sea cucumber peptides were observed using a Hitachi HT7800 Transmission electron microscope (TEM, Hitachi High-Tech Corporation, Tokyo, Japan). A Thermofisher IS50 Fourier transform infrared (FT-IR) spectrometer (Thermo Fisher Scientific Inc., Waltham, MA, USA) was employed to analyze the sea cucumber collagen peptide samples, with the spectral scanning range set at 500 cm^−1^ to 4000 cm^−1^. A TA Q600 thermogravimetric analyzer (TGA, TA Instruments Inc., New Castle, DE, USA) was used for thermogravimetric analysis of sea cucumber peptide samples, and the testing temperature range was 0 °C to 1000 °C. The circular dichroism (CD) spectra of the samples were determined using a Chirascan V100 circular dichroism spectrometer (Applied Photophysics Ltd., Leatherhead, Surrey, UK) within the wavelength range of 190–260 nm. The hydroxyproline assay kit was purchased from Nanjing Jiancheng Bioengineering Institute, Nanjing, China, and the operation was carried out according to the instructions. The proteins were identified by chemiluminescence using an ECL kit (Thermo Fisher Scientific Inc., Waltham, MA, USA), and photographs of the target bands were recorded using a Gel Imaging System (Bio-Rad Laboratories Inc., Hercules, CA, USA).

### 2.2. Extraction and Characterization Detection of Sea Cucumber Collagen Peptides

1000 g of fresh sea cucumbers were processed by removing their internal organs and longitudinal muscle layers to obtain the body walls. The body walls were then soaked in deionized water for 6 h to desalinate, and this soaking procedure was repeated three times. Distilled water was added to the body walls, which were then crushed and homogenized. The sea cucumber body walls were first sheared and broken into fine tissue particles, followed by high-speed homogenization to fully disrupt the tissue structure and release intracellular components. Papain was added at 2% of the fresh weight of the body walls, followed by shear emulsification treatment. The mixture was heated to 42 °C for enzymatic hydrolysis for 10 h, and then heated to 90 °C to inactivate the enzyme for 20 min. The enzymatic hydrolysate was centrifuged to remove impurities, and sea cucumber collagen peptides were obtained through low-temperature freeze-drying. The sea cucumber collagen peptides were chemically characterized by determining their total protein, hydroxyproline, and collagen contents, as well as their peptide profiles, following established methods [34].

### 2.3. Experimental Animals

C57BL/6 mice, male, were housed in the SPF-grade barrier system at Dalian Medical University. The mice were randomly divided into 3 treatment groups, with 10 mice in each group. During the breeding period, normal diet and 12 h light/dark cycle were ensured. All experimental protocols have been approved by the Experimental Animal Ethics Committee of Dalian Medical University (NO. AEE24022).

### 2.4. Aging Mice and Experimental Grouping

Excessive D-galactose accumulates in vivo and induces oxidative stress and the formation of advanced glycation end products, resulting in oxidative damage, inflammation, and accelerated skin aging. Therefore, the D-galactose-induced aging model is widely used to evaluate anti-aging effects [35]. To establish the aging model, 8-week-old mice were subcutaneously injected with 5% D-galactose at a dose of 1000 mg/kg daily. The normal control group, also known as the young group, received daily subcutaneous injections of saline. After 42 consecutive days of injection, the experiment was assigned to three groups: control group, aging group with D-galactose, and treatment group with sea cucumber polypeptides, with 10 mice in each one. The control group and aging group were given distilled water by gavage once a day, while the treatment group was given sea cucumber polypeptides by gavage at a dose of 2 g/kg·d. The gavage volume/body weight was the same for the control group, the aging group, and the collagen peptides treatment group. The mice were weighed daily, and all were gavaged for 30 consecutive days prior to the end of the animal experiment. The mice were anesthetized and dissected for sampling.

### 2.5. Skin Barrier Testing and Skin Moisture Content Detection

Barrier detection instruments were used to test the skin of mice. Before testing, each mouse’s right back was shaved using a shaver, and indicators such as skin barrier were recorded. To detect the moisture content of mouse skin using a dryer, the mice were shaved, and a piece of skin approximately 2 cm × 2 cm in size was taken from the middle of their back. The skin was weighed, baked in a 65 °C oven for 24 h, and then weighed again. By calculating the weight difference in the skin sample before and after baking, the moisture content was determined.

### 2.6. Hydroxyproline Content Detection

The content of hydroxyproline in tissues, serum, urine, and feces was determined using the alkaline hydrolysis method. The collected urine, serum, feces, and skin tissues (freshly frozen) of mice were hydrolyzed, with the pH value adjusted to 6.0–6.8. Activated charcoal was added, and the samples were centrifuged at 3500 rpm. The supernatant was removed, and the absorbance values of each tube were measured at a wavelength of 550 nm.

### 2.7. H&E Staining and Immunofluorescence

H&E staining is a fundamental histological technique used to visualize tissue architecture and cellular morphology. Hematoxylin stains cell nuclei blue-purple, while eosin stains the cytoplasm and extracellular matrix pink, providing contrast that allows for the assessment of structural changes including epidermal thickness, dermal collagen organization, and inflammatory cell infiltration to evaluate histological differences among treatment groups. The skin of the mice was shaved, and the skin near the back of the neck was excised and fixed in 10% formalin for 48 h. The tissue was dehydrated, paraffin-embedded, and cut into 5-μm-thick sections, and observed for structural morphological changes using H&E staining. For paraffin sections, they were first dewaxed and hydrated, followed by high-temperature and high-pressure antigen retrieval. Triton X-100 (Sigma-Aldrich, St. Louis, MO, USA) was used to permeabilize the cell membrane, while goat serum was employed to block nonspecific binding sites, and primary antibodies against GRP78 and ATF6 were incubated overnight. The cell nuclei were labeled with the fluorescent dye DAPI, and the samples were observed under a fluorescence microscope within 2 h. Images were collected and analyzed.

### 2.8. Scanning Electron Microscopy of Skin Tissue

The mice were shaved, and the skin near the back of the neck was excised and cut into small pieces (2 mm × 3 mm). The tissues were fixed in 2.5% glutaraldehyde for 48 h, dehydrated with gradient alcohol, dried using a carbon dioxide critical point dryer, and imaged with TEM.

### 2.9. Western Blot

Total protein was extracted from skin using commercially available kits. Relative protein quantification was performed using BCA. Equal amounts of each sample (50 μg) were run on SDS-PAGE and transferred to PVDF membranes. PVDF membranes were blocked with 5% skim milk powder at room temperature for 2 h, washed 3 times with TBST, and then incubated overnight at 4 °C with diluted primary antibodies. The antibodies against GRP78, ATF6, ATF4, PERK, p-PERK, P53 and GAPDH were incubated overnight as primary antibodies followed with secondary antibodies. Finally, the proteins were identified by chemiluminescence using an ECL kit, and photographs of the target bands were recorded using a Gel Imaging System. The grayscale values of the blotting bands were calculated using Image J software (Version 1.53t).

### 2.10. Statistical Analyses

Data were expressed as Mean ± SD. Statistical analysis of the data was conducted using a two-tailed unpaired Student’s *t*-test, implemented in GraphPad Prism 9.0. A statistically significant difference was indicated when * *p* < 0.05.

## 3. Results

### 3.1. Extraction and Characterization Analysis of Sea Cucumber Collagen Peptides

**Figure 1:**
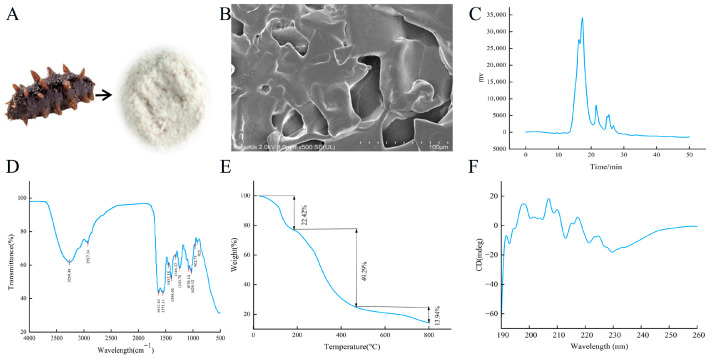
Characterization analysis of sea cucumber collagen peptides. (**A**) Sea cucumber and sea cucumber collagen peptides sample; (**B**) TEM images of sea cucumber collagen peptides; (**C**) HPLC analysis of sea cucumber collagen peptides; (**D**) FTIR spectrum analysis; (**E**) Thermogravimetric analysis; (**F**) Circular dichroism analysis.

According to the experimental method, the sea cucumber collagen peptides extracted from sea cucumbers (Figure 1A) were analyzed by HPLC. The results showed that the molecular weights of the extracted sea cucumber collagen peptides were mainly distributed between 250 and 1000 Da (Figure 1C). Among them, the collagen peptides with a size of 250–500 Da accounted for 54%, and those with a size of 500–1000 Da accounted for 42%. This indicates that the collagen peptides are mainly small molecular peptides, which facilitate absorption by body tissues and enable them to exert their functions. The body wall of sea cucumber contained type I collagen, which accounted for up to 70% of the total protein. Collagen featured a helical domain formed by repeated Gly-X-Y amino acid sequences, and hydroxyproline served as the characteristic amino acid of collagen. The sea cucumber collagen peptides prepared via enzymatic hydrolysis contain typical peptide fragments such as the Pro-Hyp dipeptide. The total protein content of the sample was determined to be 67.9 g/100 g, with a hydroxyproline content of 3.51 g/100 g. The collagen content calculated from hydroxyproline was 38.96 g/100 g, accounting for 57.38% of the total protein. Furthermore, 488 peptides were identified by mass spectrometry, among which 127 peptides contained the P-H sequence, accounting for 26.02% of the total identified peptides. The protein description of these 127 peptides was all correlated with collagen, which verified and identified the properties of the sea cucumber collagen peptides.

The TEM image of the sea cucumber peptide sample was shown in Figure 1B, from which the fibrous network characteristics of sea cucumber peptides could be observed. As presented in Figure 1D, an absorption peak at 3269.48 cm^−1^ corresponded to the stretching vibration of N-H, which was consistent with the characteristics of peptide substances; an absorption peak at 2927.34 cm^−1^ indicated the presence of saturated hydrocarbon structures in sea cucumber peptide molecules, such as methyl and methylene groups in amino acid side chains; and an absorption peak near 1632.62 cm^−1^ originated from the stretching vibration of the secondary structure of peptide chains. An absorption peak near 1551.11 cm^−1^ fell within the absorption range of N-H bending vibration and C-N stretching vibration, confirming the existence of peptide bonds in sea cucumber peptides; multiple absorption peaks existed at 1316.22 cm^−1^, 1243.78 cm^−1^, 1119.01 cm^−1^, and this region included the stretching vibration absorption of various single bonds such as C-O and C-N, which could be derived from the C-N bonds in peptide bonds and the C-O bonds formed by hydroxyl groups in amino acid side chains with other atoms, reflecting the complexity of the molecular structure of sea cucumber peptides. Through the analysis of each characteristic absorption peak in the infrared spectrum, it could be confirmed that the sample had the typical structural characteristics of peptide substances.

The TGA curve in Figure 1E displayed the mass change in the sample at different temperatures, enabling better analysis of the thermal stability of sea cucumber peptides. The weight loss of sea cucumber peptides occurred in three stages: the first stage was 0–200 °C, where the weight loss was mainly caused by the evaporation of bound water and adsorbed water, with a weight loss rate of 22.42%; the second stage was 200–400 °C, which showed the maximum weight loss and represented the main thermal decomposition stage of sea cucumber peptides. During this stage, peptide bonds and side chain groups in sea cucumber peptide molecules broke, and macromolecules decomposed into small molecules and volatilized, resulting in a significant mass decrease, with a weight loss rate of 49.29%; the third stage (400–800 °C) corresponded to high-temperature carbonization, with a weight loss of 13.94% and ash as the main residue. The mass loss rate was lower than that in the second stage, indicating higher thermal stability of the second stage residue. CD spectroscopy is mainly used to determine the secondary structure of proteins. After enzymatic hydrolysis, the sea cucumber collagen peptides had small molecular weight and loose structure, mainly in the form of random coils (Figure 1F).

### 3.2. Detection of Skin Barrier Function, Moisture Content, and Hydroxyproline Content

The skin barrier is composed of the stratum corneum, lipids, and epidermis. Changes in these structures can alter the skin resistance/conductance values. Therefore, we utilized non-invasive skin barrier function detection technology to assess the skin barrier function. This method is convenient, rapid, and most importantly, non-invasive for evaluating skin barrier function. The detection began with gavage and was performed once a week. The results showed that the skin barrier score of the aging group mice was markedly lower than that of the control group (*p* < 0.05).

**Figure 2:**
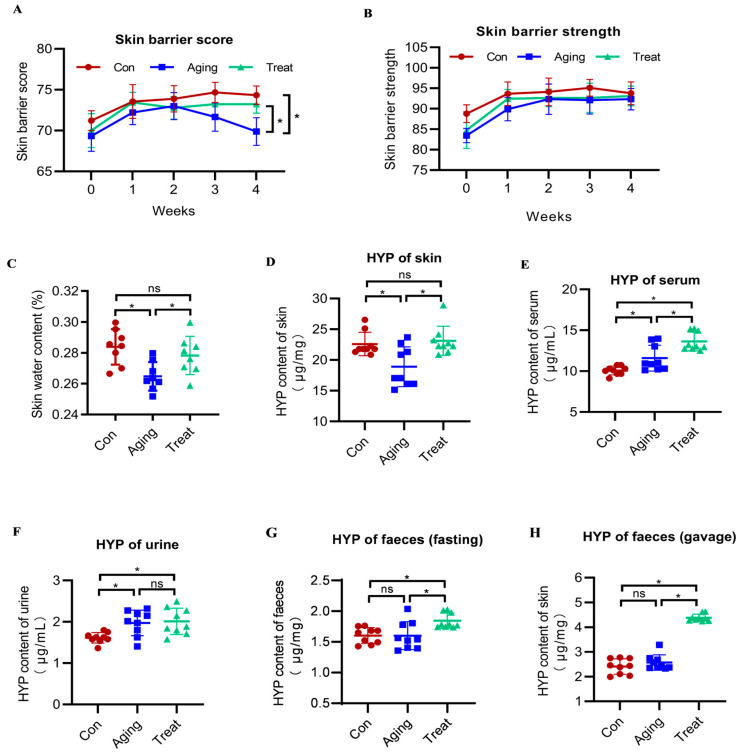
The barrier function, the moisture content, and the hydroxyproline content of the skin. (**A**) Skin barrier score of mice skin of each group; (**B**) Skin barrier strength of mice skin of each group; (**C**) Skin water content of each group mice; (**D**) HYP of skin of each group; (**E**) HYP of serum; (**F**) HYP of urine; (**G**) HYP of feces with fasting for 12 h of each group mice; (**H**) HYP of feces after gavage 2 h. * *p* < 0.05, ns: no significance.

The skin barrier score is a comprehensive evaluation index that combines skin barrier strength and skin moisture content. The skin barrier strength is a direct measured value of the skin barrier’s function. However, starting from the third week of gavage, the skin barrier score of the treatment group was significantly higher than that of the aging group (*p* < 0.05) and showed no significant difference from the control group (Figure 2A). As for the skin barrier strength, the treatment group showed a slight improvement trend, but there was no significant difference among the three groups of mice (*p* > 0.05). The skin barrier score clearly indicated that skin function was impaired in aging mice, and treatment with sea cucumber polypeptides significantly improved the barrier score in aging skin. Skin barrier strength reflects the status of the stratum corneum. The results of skin barrier strength demonstrated that neither aging nor sea cucumber polypeptide treatment exerted significant effects on the stratum corneum within the experimental period.

The skin moisture content can help assess skin health. Through statistical analysis of the experimental results of measuring skin moisture content in mice using the drying method, it was found that the skin moisture content in the aging group mice was significantly lower than that in the control group mice (*p* < 0.05). Administration of sea cucumber polypeptides by gavage could significantly increase the skin moisture content in aging mice, restoring it to a level that was indistinguishable from the control group (*p* > 0.05) (Figure 2C).

Additionally, to evaluate the absorption and metabolism of collagen peptides in vivo, we measured the hydroxyproline content in skin, serum, urine, and feces before and after gavage. The results indicated that the hydroxyproline content in the skin of aging mice was substantially lower than that of the control group (*p* < 0.05), while sea cucumber collagen peptides significantly increased the hydroxyproline content in the skin of mice (*p* < 0.05) (Figure 2D). Hydroxyproline content in mouse serum differed across groups, with that of aging mice being significantly higher than the control group (*p* < 0.05). However, the serum hydroxyproline content of mice in the sea cucumber peptides gavage group was significantly increased compared to both the control group and the aging group (*p* < 0.05) (Figure 2E). Urine hydroxyproline detection results showed that the urine hydroxyproline content of aging mice was significantly higher than the control group (*p* < 0.05), while gavage with sea cucumber collagen peptides did not increase the urine hydroxyproline content of mice (*p* > 0.05) (Figure 2F). Fecal hydroxyproline content was measured in mice that had fasted for 12 h and in mice that had been fed ad libitum and then had been gavaged for 2 h. It was found that no significant difference in hydroxyproline content existed between the normal control and aging groups under fasting conditions (*p* > 0.05), while fecal hydroxyproline content in the sea cucumber peptide gavage group was significantly higher than that in the control and aging groups under the same conditions (*p* < 0.05) (Figure 2G). After 2 h of gavage with sea cucumber collagen peptides, there was no significant difference in fecal hydroxyproline content between the normal control group and the aging group (*p* > 0.05), but the fecal hydroxyproline content of mice in the gavage group was significantly higher than that of the control group and the aging group (*p* < 0.05) (Figure 2H). These hydroxyproline detection results indicated that gavage with sea cucumber collagen peptides reflected the absorption and excretion of collagen peptides in the body, especially the metabolic status of collagen in the skin.

### 3.3. Effect of Sea Cucumber Collagen Peptides on Collagen Fibers

**Figure 3:**
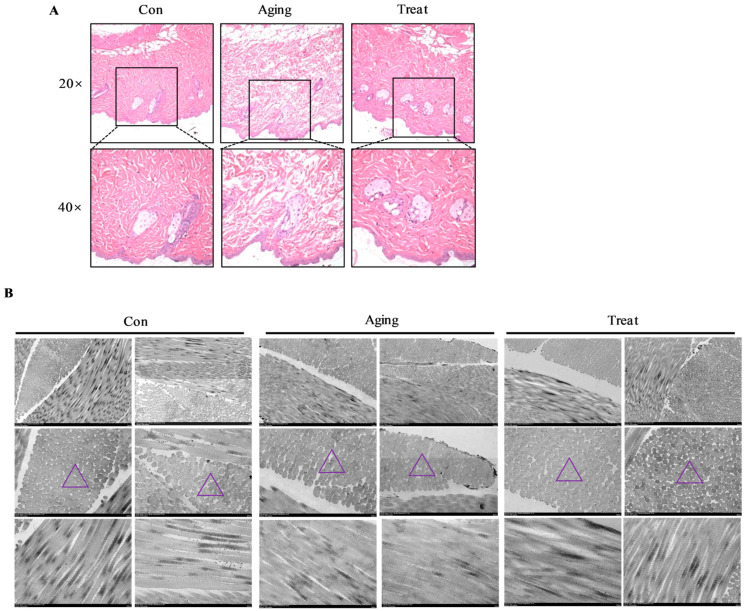
H&E of skin and TEM of collagen fiber. (**A**) H&E of skin of each group; (**B**) TEM of skin collagen fiber of each group (Hitachi HT7800, scale bar = 500 nm). Triangles represent the cross-sections of collagen fibers.

To further investigate the impact of sea cucumber polypeptides on skin tissue structure, H&E staining was employed to observe the morphology of skin tissue. The results indicated that, compared to the control group, the collagen fiber structure of the skin in the aging group was loose. In contrast, the skin structure of the mice in the sea cucumber polypeptides treatment group was more defined and dense, with a significant increase in collagen content (Figure 3A). Skin scanning electron microscopy provided a more microscopic observation of skin collagen fiber tissue, yielding results consistent with H&E staining. Notably, the electron microscopy images of collagen fiber cross-sections revealed that the collagen fibers in the aging group were loosely arranged compared to the normal control group, whereas the collagen arrangement in the skin after sea cucumber polypeptides treatment was more compact (Figure 3B). This suggested that sea cucumber polypeptides increased the collagen fiber content in aging skin tissue.

### 3.4. Effect of Sea Cucumber Collagen Peptides on Fibroblasts and Prickle Cells

**Figure 4:**
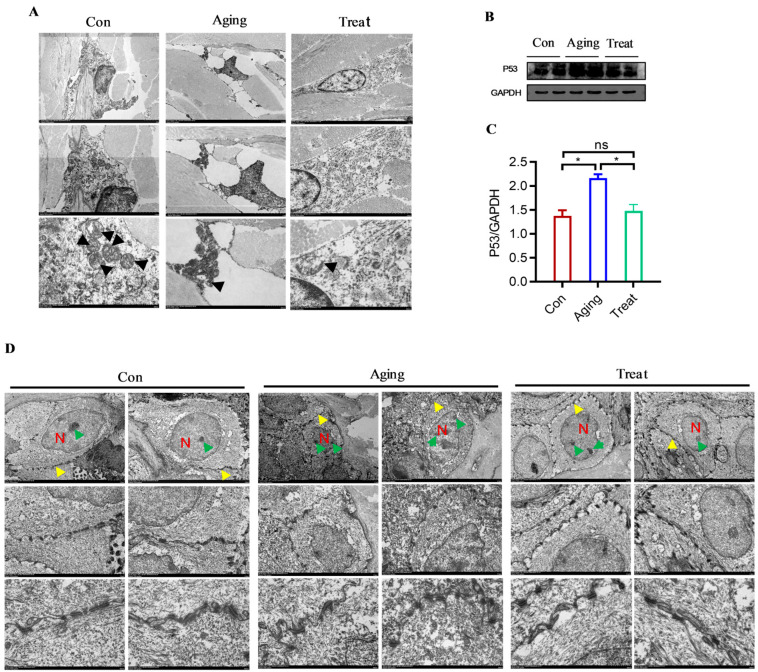
TEM of fibroblasts, prickle cells and desmosome connections of skin. (**A**) Ultra microscopic structure of skin fibroblasts from TEM (Hitachi HT7800, scale bar = 2.0 μm); (**B**) Western Blot of p53; (**C**) Gray value analysis of P53/GAPDH; (**D**) Ultra microscopic structure of prickle cells and desmosome connections from TEM (Hitachi HT7800, scale bar = 2.0 μm). * *p* < 0.05, ns: no significance. N: nucleus; Green arrows: nucleoli; Yellow arrows: desmosome junctions.

Observations on fibroblasts using scanning electron microscopy revealed that cells in the normal control group exhibited irregular morphology, multiple protrusions, enlarged nuclei, regular and complete nuclear membranes, prominent and multiple nucleoli, abundant ER with regular structure, where tubular and flattened ER connected to form a membrane-like tubular network system, abundant mitochondria with clear cristae structure, but disordered mitochondrial cristae arrangement. The ER structure was also disordered, with enlarged ER cavities appearing vacuolar. The skin fibroblasts in the sea cucumber polypeptides treatment group were larger, irregular in shape, with multiple protrusions, enlarged nuclei, regular nuclear membrane morphology, prominent nucleoli, single or multiple nucleoli, fewer mitochondria (compared to the control group), clearer mitochondrial cristae structure (compared to the aging group), and richer and more regular ER structure (Figure 4A). These structural changes indicated that the skin fibroblasts of the aging group mice exhibited aging-related manifestations, while sea cucumber polypeptides could effectively delay or combat fibroblast aging and improve cell status. Subsequently, Western blot analysis of skin tissues was conducted to detect aging-related markers. The results revealed that the expression of P53 in the aging group skin was remarkably higher than that in the control group and treatment group (Figure 4B,C) (*p* < 0.05). As a key regulator of cell senescence and apoptosis, elevated P53 expression is closely associated with fibroblast senescence, impaired proliferation, and accelerated skin aging. The significantly reduced P53 level in the treatment group suggested that sea cucumber collagen peptides could inhibit abnormal overexpression of P53, alleviate fibroblast senescence and apoptosis, maintain the normal function of dermal fibroblasts, and thus delay skin aging.

The status of fibroblasts is closely related to other skin tissue cells. Therefore, we observed the skin prickle cells and desmosomal junctions under electron microscopy. The results showed that the nuclei of prickle cells in the control group were round or oval, with a single round nucleolus located centrally in the cell. The desmosomal junctions were tight and regular. In the aging group, the nuclei of skin tissue prickle cells exhibited irregular morphology, with nuclear membranes shrinking and appearing protruding or concave. Multiple nucleoli were located at the edge of the nucleus, with irregular shapes. Although the desmosomal junctions were tight, they were relatively disordered. In the treatment group, the nuclei of skin prickle cells were relatively regular, round or oval; multiple nucleoli were regular in shape, located closer to the nuclear membrane, and the desmosomal junctions were tight and regular (Figure 4D). Surprisingly, we have discovered the positive effects of sea cucumber collagen peptides on the stratum spinosum layer, providing new ideas and means for improving skin barrier function.

### 3.5. Effect of Sea Cucumber Collagen Peptides on ER Stress in Senescent Skin Fibroblasts

**Figure 5:**
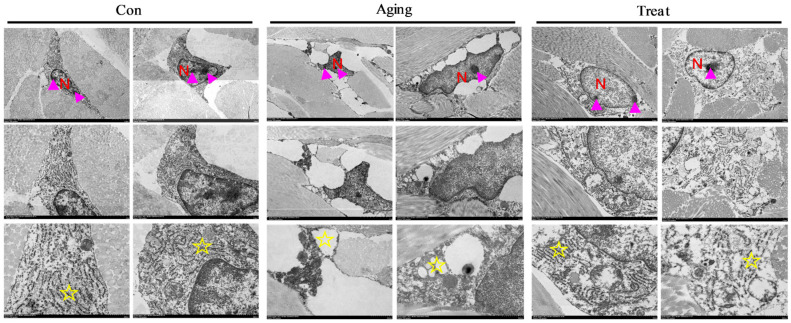
Ultra microscopic structure of fibroblasts ER (Hitachi HT7800, scale bar = 2.0 μm). N: nucleus; Pink arrows: nucleoli; Yellow pentagrams: endoplasmic reticulum.

To further explore the mechanism of action of sea cucumber polypeptides on fibroblasts, we focused on the ultrastructure of fibroblasts. Electron microscopy results indicated significant ER structural disorder in skin fibroblasts of the aging group, with the ER lumen enlarged and appearing vacuolar. However, after treatment, the ER stress was significantly inhibited (Figure 5), and the morphology of the ER was significantly improved.

**Figure 6:**
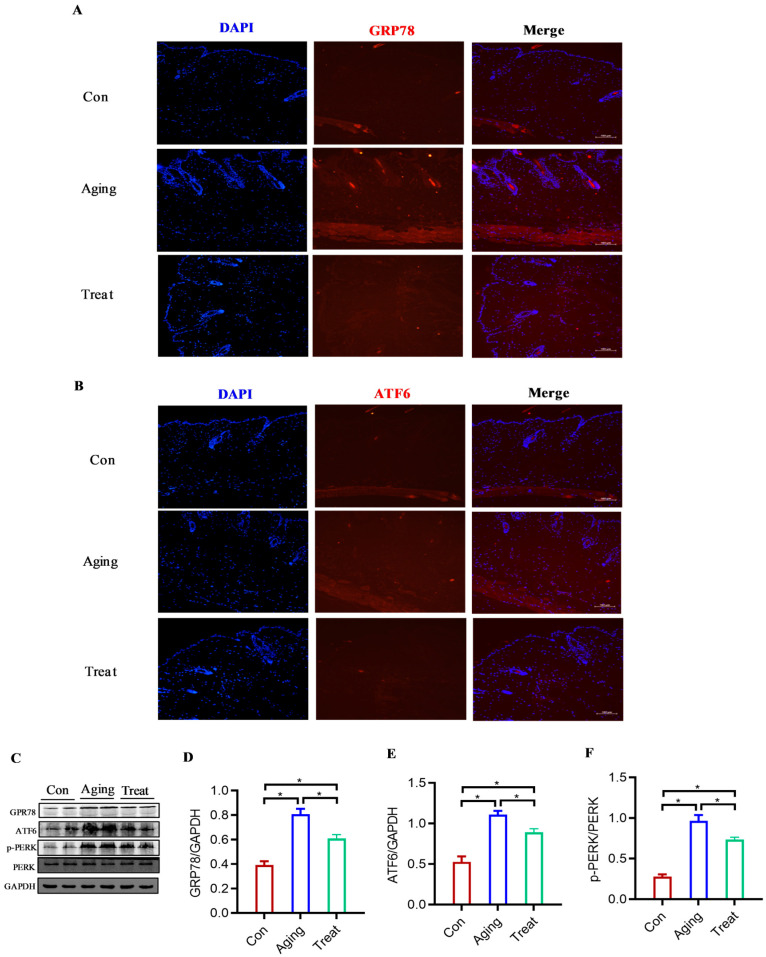
Detection of ER stress molecules. (**A**) Immunofluorescence of GRP78 of skin tissue; (**B**) Immunofluorescence of ATF6 of skin tissue; (**C**) Western Blot of GRP78, ATF6, p-PERK and PERK; (**D**) Gray value analysis of GRP78/GAPDH; (**E**) Gray value analysis of ATF6/GAPDH; (**F**) Gray value analysis of p-PERK/PERK. * *p* < 0.05. scale bar = 100 μm

We used immunofluorescence to detect the marker molecules of ER stress, GRP78 and ATF6, and found that the expression levels of GRP78 and ATF6 in the aging group were significantly increased compared to the control group, while the expression of GRP78 and ATF6 in the polypeptides-treated group of mice skin was significantly reduced (Figure 6A,B). Further verification of the expression of GRP78, ATF6, and PERK molecules using Western Blot revealed that sea cucumber polypeptides significantly reduced the expression of GRP78 and ATF6 in mouse skin, as well as the level of phosphorylated PERK protein (Figure 6C). Grayscale analysis showed a consistent trend (Figure 6D–F). These results suggested that the anti-aging effect of sea cucumber polypeptides on the skin was closely related to improving the ER status of fibroblasts.

## 4. Discussion

As individuals age, skin aging becomes prominent with visible symptoms and functional alterations, yet relevant research remains limited due to the complexity of skin cells and tissue structure, and the underlying mechanism as well as effective anti-aging interventions are still unclear. Collagen peptides show potential in delaying skin aging, but their specific mode of action and systemic benefits require further investigation [36]. This study extracted collagen peptides from a unique sea cucumber species in the sea area of Dalian, China, and performed in vivo experiments in mice. Sea cucumber collagen peptides were small peptides obtained by hydrolyzing sea cucumber proteins [37]. Compared with collagen peptides derived from porcine or bovine sources, sea cucumber peptides offer unique advantages, including a lower risk of zoonotic disease transmission, broader dietary and religious compatibility [38,39]. In the present study, physicochemical characterization was performed on the prepared sea cucumber collagen peptides to confirm their high proportion of effective collagen peptide components. Skin barrier function in experimental mice was subsequently evaluated; the results demonstrated that oral administration of collagen peptides significantly enhanced skin barrier strength and moisture content in aging model mice. To further assess the in vivo absorption and metabolism of collagen peptides, the content of hydroxyproline, a characteristic amino acid of collagen, was determined in skin tissue, serum, urine, and feces before and after gavage. By analyzing the loss and replenishment of collagen in combination with hydroxyproline levels, this study verified that supplementation with sea cucumber collagen peptides effectively mitigated skin aging, suggesting that small-molecule collagen peptides exert beneficial effects at the cellular nutritional level in skin tissue [34]. Specifically, collagen peptide supplementation significantly increased hydroxyproline content in skin and serum while reducing its excessive loss in excreta, which directly reflected enhanced collagen synthesis and reduced degradation in skin tissue. From the perspective of skin barrier, the improvement of skin moisture content and barrier strength further confirmed the protective effect of collagen peptides on skin structure and function. These results are consistent with previous studies, indicating that collagen peptides can effectively maintain dermal structural integrity, enhance skin water retention and moisturizing function, and delay the aging process of skin tissue by improving the nutritional microenvironment of skin cells [40].

At the tissue level, this study investigated the effects of sea cucumber collagen peptides on skin tissue structure by H&E staining, and revealed that collagen peptide treatment resulted in a denser arrangement of dermal collagen fibers. At the cellular level, sea cucumber collagen peptides ameliorated mitochondrial morphology in senescent skin fibroblasts, as well as the status of prickle cells and desmosome junctions, thereby exerting anti-skin-aging effects. Dermal fibroblasts are the core cells responsible for the structure and function of the skin, undertaking various crucial tasks such as synthesizing and maintaining the skin matrix, participating in wound healing, regulating immune responses, and maintaining hydration [41]. With aging, the function of fibroblasts gradually declines, resulting in skin aging, reduced elasticity, and impaired repair capacity. As long-lived cells prone to accumulating age-related damage [42], skin fibroblasts are regarded as potential amplifiers of skin aging and inflammation [9]. Therefore, investigating and improving fibroblast function, especially through anti-aging treatments, may provide effective intervention strategies for skin aging [43]. Consequently, our research primarily focused on the changes in fibroblasts. Fortunately, we did observe the effects of sea cucumber collagen peptides on fibroblasts. Previous studies have found that sea cucumber collagen peptides play a key role in anti-aging, immune regulation, and antioxidant effects [44]. Sea cucumber collagen peptides slow down the aging process by inhibiting oxidative stress, promoting cell repair, and regulating the immune system. Research has shown that sea cucumber collagen peptides can delay cell aging and promote skin repair and regeneration by regulating intracellular signaling pathways [45]. They also regulate the secretion of cytokines including TNF-α and IL-6 [46], thereby strengthening disease resistance. Bioactive compounds from sea cucumbers exert anti-fatigue effects via regulating the NRF2/ARE and AMPK/PGC-1α signaling pathways [47]. In addition, they directly act on tumor cells, inducing cell cycle arrest at the DNA synthesis (S) phase, inhibiting proliferation, and promoting apoptosis [48]; they can also improve anti-tumor capacity by activating the immune system. As reported by Bismoy Mazumder, sea cucumber extracts protect skin cells during wound healing by shielding organelles from oxidative damage and modulating glycolysis/gluconeogenesis as well as endoplasmic reticulum protein processing pathways [49]. Consistently, the enhancement of skin barrier function by sea cucumber collagen peptides was linked to an improvement in the condition of keratinocytes, suggesting that restoring the state of these aging cells may underlie the observed benefits for skin immune and barrier functions [50]. Collectively, the results of this study further confirmed that sea cucumber collagen peptides could exert remarkable anti-skin aging effects by remodeling dermal tissue structure and improving the physiological function of aging skin fibroblasts.

To further explore the mechanism of action of sea cucumber collagen peptides on skin fibroblasts, this study focused on the ultrastructure of these cells. Electron microscopy results revealed that skin fibroblasts in the aging group exhibited obvious structural disorders of the ER, with dilated ER lumens appearing vacuolated. However, after treatment with sea cucumber collagen peptides, ER stress was significantly inhibited, and the morphology of the ER was markedly improved. ER stress induces a response through three major UPR pathways: IRE1, PERK, and ATF6. However, when stress is excessive, the activation of UPR can induce cell aging, death, or dysfunction, and altering UPR signaling in animal models can reduce the pathological consequences of aging [51]. ER stress can trigger oxidative stress and excessive free radical production, which damage cellular lipids, proteins, and DNA, thereby accelerating the aging process. ER stress can activate the p53 protein [52], a classic tumor suppressor typically stimulated by DNA damage or cell death signals. Activated p53 further induces cell cycle arrest, apoptosis, or cellular senescence, thus promoting aging [53]. In this study, Western blot analysis further demonstrated that collagen peptide treatment downregulated the expression of the aging marker protein P53. Given the involvement of ER stress in aging, numerous studies have focused on regulating the ER stress response to delay aging. Antioxidants such as resveratrol [54] and NAD+ precursors can alleviate oxidative damage caused by ER stress and may exert anti-aging effects. In addition, small-molecule agents, such as XBP1 agonists and PERK inhibitors, are being investigated to modulate ER stress and attenuate age-related diseases. Moreover, crosstalk exists between ER stress and autophagy [55]. Promoting the proliferation of dermal fibroblasts represents an effective strategy against skin aging. Research has demonstrated that regulating autophagy and enhancing fibroblast proliferation can exert skin anti-aging effects [14]. Nevertheless, studies on inhibiting ER stress in senescent dermal fibroblasts to counteract aging remain limited. In this study, sea cucumber collagen peptides had an inhibitory effect on ER stress in skin fibroblasts, which might improve cellular protein homeostasis and metabolism. At a deeper level, the mechanisms through which sea cucumber collagen peptides act on ER stress via specific molecules or pathways remain unknown. Identifying the target sites of sea cucumber collagen peptides is also an issue that requires further attention in future research. Furthermore, an immunofluorescence assay was employed in this study to detect the expression of ER stress marker molecules, GRP78 and ATF6. Western blot analysis further verified the protein levels of GRP78, ATF6, and PERK. The results demonstrated that sea cucumber collagen peptides could effectively reduce the expression of GRP78 and ATF6, as well as the level of phosphorylated PERK in mouse skin. Collectively, these findings indicated that the anti-skin aging effects of sea cucumber collagen peptides are closely associated with the improvement of ER status and function in fibroblasts.

Mitochondrial dysfunction is a crucial hallmark in the aging process [56]. As individuals age, mitochondrial function gradually declines, leading to reduced ATP synthesis, increased oxidative stress, and higher production of reactive oxygen species (ROS), which accelerates cell damage and the aging process [57]. Furthermore, we have observed the impact of sea cucumber collagen peptides on the mitochondria of fibroblasts [58], and we hypothesize that the effects of sea cucumber collagen peptides on mitochondria may also be related to the ER [59]. Conversely, their impact on ER stress may also be achieved by improving mitochondria. Regardless, ER-mitochondrial communication is a pivotal aspect in the study of aging and related anti-aging drugs, potentially providing new theoretical insights into aging research.

**Figure 7:**
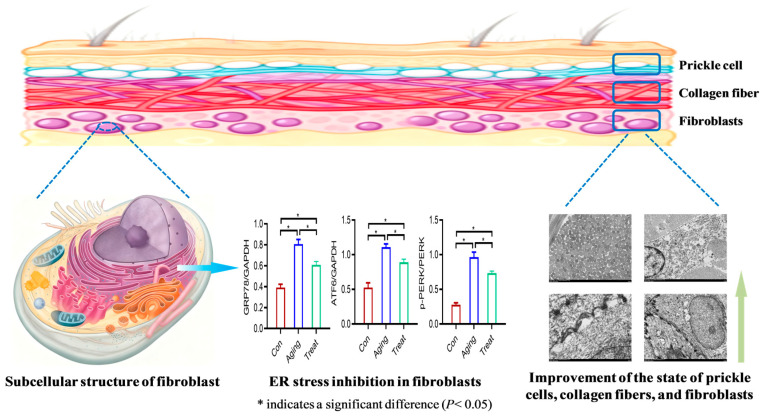
The effect of sea cucumber collagen peptide on endoplasmic reticulum stress in aging skin fibroblasts.

The structural characteristics of skin tissue, with their relatively complex composition, pose certain technical challenges for our research on aging skin. There are various types of skin cells, and fibroblasts exhibit heterogeneity [60], a characteristic that poses challenges for research. Our current experiments have not been able to effectively distinguish between different cell populations in the skin. Therefore, the total protein extracted from skin tissue encompasses proteins from various cell groups in the skin. The results of Western blotting can only reflect the level of this protein in the skin, but not the protein level of any specific cell group. To better address this issue, most of our experimental results were achieved through histopathological techniques, including scanning electron microscopy to observe the structure of organelles and immunofluorescence to detect protein expression levels. We further aim to isolate different cell populations through techniques such as single-cell sequencing or flow cytometry in the future, allowing for more precise detection of protein and gene expression in fibroblasts, prickle cells, and other cells. Collectively, the sea cucumber polypeptides could enhance the barrier function of the skin of aging mice, increase skin moisture content, improve the state of skin fibroblasts and prickle cells, and increase collagen fiber content and desmosome connections. Moreover, the findings further indicated that ER stress-related molecules are likely key targets of sea cucumber collagen peptides, with the endoplasmic reticulum itself serving as a primary organelle mediating their bioactive effects. Specifically, the mechanism underlying the anti-skin aging effect of these peptides may be associated with their dual actions: inhibiting ER stress in skin fibroblasts and improving prickle cell function. These results provided direct experimental evidence that sea cucumber collagen peptides would alleviate fibroblast ER stress, thereby supporting their potential role in ameliorating skin aging (Figure 7). The development of effective drugs to alleviate the ER stress in dermal fibroblasts has brought great hope for skin anti-aging research, and sea cucumber collagen peptides are also expected to become important ingredients of food-drug homology for skin anti-aging.

## 5. Conclusions

In conclusion, this research indicated that sea cucumber polypeptides could enhance the barrier function of the skin of aging mice, increase skin moisture content, improve the state of skin fibroblasts and prickle cells, and increase collagen fiber content and desmosome connections, thereby exerting a delaying effect on skin aging. The mechanism behind this effect may be related to their inhibition of ER stress in skin fibroblasts and improvement of the state of prickle cells. These research findings provide valuable insights into the anti-aging effects of sea cucumbers, explore the mechanisms and methods of anti-aging in fibroblasts, especially in skin beauty and maintaining a youthful state, and offer promising strategies for developing new anti-skin aging products using sea cucumber collagen peptides as core functional food ingredients.

## Data Availability

The original contributions presented in the study are included in the article, further inquiries can be directed to the corresponding authors.

## Abbreviations

The following abbreviations are used in this manuscript:

ER	Endoplasmic reticulum
GAGs	Glycosaminoglycans
PGs	Proteoglycans
ECM	Extracellular matrix
MMPs	Matrix metalloproteinases
UPR	Unfolded protein response
TIMP-1	Tissue inhibitor of metalloproteinase-1
HPLC	High performance liquid chromatography
TEM	Transmission electron microscope
FT-IR	Fourier transform infrared
TGA	Thermogravimetric analyzer
CD	Circular dichroism
ROS	Reactive oxygen species
